# Factors associated with malaria infection in Honde valley, Mutasa district, Zimbabwe, 2014: a case control study

**DOI:** 10.1186/s13104-015-1831-3

**Published:** 2015-12-29

**Authors:** Noma Mugwagwa, Joseph Mberikunashe, Notion Tafara Gombe, Mufuta Tshimanga, Donewell Bangure, More Mungati

**Affiliations:** Department of Community Medicine, University of Zimbabwe, Office 3-66 Kaguvi Building, Corner 4th/Central Avenue, Harare, Zimbabwe; Ministry of Health and Child Care, Harare, Zimbabwe

**Keywords:** Malaria, Case control study, Indoor residual spraying, Zimbabwe

## Abstract

**Background:**

In January of 2014, an outbreak of malaria was declared in the Honde Valley region of Mutasa District in Zimbabwe. The area has a hot, temperate climate and high rainfall pattern ideal for vector breeding and malaria transmission. Gravity fed irrigation channels span the valley creating a number of breeding sites for malaria vectors, mosquitoes. Malaria outbreaks have been a common occurrence in the district over the past few years despite the district meeting set targets for prevention interventions like indoor residual spraying (IRS) with pyrethroids and long lasting insecticidal nets distribution. The objectives of this study were to describe the outbreak by person, place and time, to assess the community’s knowledge on malaria transmission, signs and symptoms and treatment and to tease out factors associated with malaria infection in the district.

**Methods:**

An unmatched case–control study was carried out. Interviewer guided questionnaires were administered to residents of the valley who met the inclusion criteria in order to tease out possible factors associated with malaria infection. A case was defined as a resident of Honde Valley with a history of malaria symptoms and a confirmed diagnosis from 13 January 2014 to 26 January 2014. A control was a resident of Mutasa District who was present in the district during the time of the outbreak but did not develop symptoms of malaria. A total of 87 cases and 87 controls were enrolled.

**Results:**

Cases and controls were comparable in terms of socio-demographic characteristics and knowledge on malaria transmission, treatment and prevention. Risk factors associated with contracting malaria during the outbreak were being under the age of 5 years (OR = 9.92, CI 1, 2–80, 1), not using mosquito repellents (OR = 8, 25 CI 3, 78–18, 0), having outdoor activities before dawn and after dusk (OR = 2, 81 CI 1,04–7, 6). Having received indoor residual spraying in ones house was a risk factor for contracting malaria (OR = 1, 68 CI 0, 74–3, 83). This finding was not statistically significant. Sleeping under an insecticidal net and wearing protective clothing when outdoors after dusk were protective factors against contracting malaria (OR = 0, 27 CI 0, 12–0, 59 and OR = 0, 12 CI 0, 06–0, 25 respectively). All cases were treated according to the national malaria case management guidelines.

**Conclusions:**

Risk factors for contracting malaria were being under the age of 5 years, outdoor activities at night and not using mosquito repellents. Net use was a protective factor from contracting malaria in Mutasa District. Indoor residual spraying with pyrethroids in Honde Valley was not a protective factor against contracting malaria.

## Background

Although investments in the fight against malaria have led to significant reductions in malaria cases and deaths, the disease still is the most common vector borne disease globally. It is among the top five diseases in terms morbidity and mortality and it results in significant economic losses. It is approximated that about 3.3 billion, half the world’s population is at risk of developing malaria [1]. People living in the poorest countries are the most vulnerable to malaria. Although malaria is found in most regions of the world, mainly Asia, South America and Africa, the World Health Organisation (WHO) Africa Region is the greatest affected region of them all. World Health Organisation in the Global Malaria Report of 2013 reported that in 90 % of all malaria deaths occurred in the African region [1].

Malaria is endemic in Zimbabwe and remains one of the top five causes of morbidity and mortality in the country. The Ministry of Health and Child Care (MOHCC) through the National Malaria Control Program (NMCP) manages control of the disease in the country by using different interventions like vector control, case management and behavioural change communication. Over the past decade, the country has seen a decline in both annual malaria cases and deaths. This can be attributed to scaling up of efforts in disease prevention and control measures. It is interesting however to note that National Malaria Control Program statistics reveal that Manicaland Province, mainly in Mutasa District, has become and remains a problem zone for the country, contributing a third to national malaria cases and deaths annually. This is despite the province meeting set targets of preventative and control interventions.

During week 3 of 2014, the District Health Information Officer reported an increased number of malaria cases in health facilities across Mutasa District of Manicaland Province. Efforts on preventive interventions including IRS and LLIN distribution by the MOHCC through the NMCP, with help from health partners in 2013 had met the set targets for the district. Net distribution was at 100 % and IRS coverage was 87 % protecting 85 % of the population. Despite these successes, malaria cases were surpassing action threshold levels and malaria deaths were being recorded. This investigation set out to uncover some of the factors that are leading to continued increase malaria infections in Mutasa despite efforts in prevention and control interventions. Specifically, to describe the outbreak by person, place and time, to assess the community’s knowledge on malaria transmission, signs and symptoms and treatment and to tease out factors associated with malaria infection in the district.

## Methods

*Study Design* An unmatched 1:1 case control study was be conducted

*A case* was a resident of Mutasa District with a history of malaria symptoms and had an rapid diagnostic test (RDT) or microscopy confirmed diagnosis of malaria from 13 January 2014 to 26 January. Symptoms of malaria referred to one or a combination of fever, vomiting headache, general body malaise and rigors/chills.

*A control* was a resident of Mutasa District who was present in the district during the time of the outbreak but did not develop symptoms of malaria

*Inclusion criteria* Any individual fitting the case definition was included in the study despite age or other socio-demographic characteristics.

*Exclusion criteria* Any individual who was not in the district during the outbreak was excluded from the study. Those who had symptoms suggestive of malaria infection but had no confirmation of the diagnosis by RDT or microscopy were excluded from the study.

### Study setting

The study was carried out in Honde Valley in Mutasa District in Zimbabwe. The valley is in the eastern part of the country. It is an agricultural region with a hot temperate climate ideal for vector breeding and malaria transmission. The valley lies in the region which is one of the high malaria burdened areas.

### Study population

The study population comprised of residents of Honde Valley, Mutasa District in Zimbabwe.

### Sample size

Using Stat Calc, assuming sleeping in an unsprayed room as a significant risk factor for contracting malaria with an odds ratio of 2.53, with 36.4 % of controls having been exposed (study by Maenzanise S R et al. entitled ‘Malaria investigation in Msapakaruma, Kariba District, 2004-unpublished paper) using a power of 80 % and confidence interval of 95 %—The calculated sample size was 88 cases and 88 controls. However, 87 cases and 87 controls were interviewed.

### Sampling procedure

Four out of the six affected health facilities were purposively included in the study. Cases were sampled from each of the four health facilities proportionately.

The first case from each facility was randomly selected using the lottery method. Subsequently, from line list created at each of the health facility, depending on number of cases the health facility was proportionately contributing to the study every tenth name on list was enrolled in the study. A neighbour of a case who fits the definition of a control was used as a control.

### Data collection

Data was collected using interviewer guided questionnaires.

### Data analysis

Quantitative data was captured and analysed using Epi—info version 3.5.1™. The statistical software was also used to generate frequencies and means and calculate odds ratios and their corresponding 95 % confidence intervals. Qualitative data was quoted and reported as said by participants.

### Permission and ethical considerations

Permission to carry out the study was sought from and granted by the Health Studies Office in the Ministry of Health and Child Care, Provincial Medical Director of Manicaland Province and the District Medical Officer of Mutasa District.

The aim of the study was fully explained to all participants and guardians of participants and written informed consent was sought from all participants and guardians of minors included in the study. Assent was obtained from the all minors. Permission to take pictures was sought from the participants. Confidentiality was assured and maintained throughout the study.

## Results

An increase of malaria cases was recorded in Mutasa District in the third and fourth week of 2014. A total of 4755 malaria cases were seen between 13 January and 26 January 2014. This gives an incidence ratio of 28 cases/1000 population for the district (population of Mutasa District was 169,000 as at 2012 census). Approximately 88 % (4184/4755) of these cases were from Honde Valley (Mutasa North) area.

The case control ratio was a 1: 1. A total of 87 cases and 87 controls were interviewed. Table 1 shows the demographic characteristics of the study participants.

**Table 1:** Socio-demographic characteristics of study participants in Honde Valley, Zimbabwe, 2014

Factor	Category	Cases n (%)	Controls n (%)	*p* value
Sex	Female	39 (44, 8)	48 (55, 2)	0.172
	Male	48 (55, 2)	39 (44, 8)	
Marital status	Married	51 (58, 6)	55 (63, 2)	0.472
	Single	36 (41, 4)	32 (36, 8)	
Monthly income	<$100	75 (86, 2)	72 (82, 8)	0.539
	>$100	12 (13, 8)	15 (17, 2)	
Education	None	16 (18, 4)	6 (6, 89)	0.023
	Some education	71 (81, 6)	81 (93,1)	
Occupation	Farmer	82 (94, 3)	78 (89, 7)	0.265
	Other	5 (5,7)	9 (10, 3)	
Religion	Apostolic	44 (50, 6)	48 (55, 1)	
	Other	43 (49, 4)	39 (44, 8)	0.543
Age	Median (Q1, Q30)	28 (15; 38)	32 (21; 37)	0.327

Cases and controls were comparable in terms of age, sex, marital status, occupation and religion. There was however a significant difference between cases and controls in terms of education. Most of the cases from the valley were noted to come from settlements near water bodies and wetlands. Also affected were estate and tea factory workers who were on duty during outbreak weeks. Some cases were noted to come from across the Zimbabwe–Mozambican boarder.

**Fig. 1:**
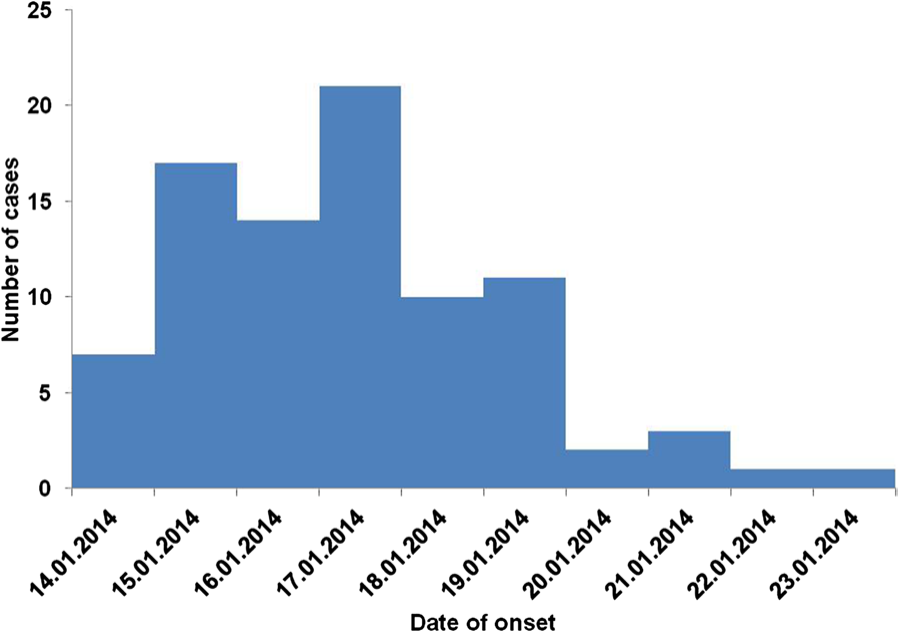
Epidemic curve—malaria outbreak in Honde Valley, Mutasa District, Zimbabwe, 2014

The epidemic curve is based on date of onset of clinical symptoms for malaria cases included in the study. Malaria cases started increasing from the 15th of January and a peak was noted on the 17th January. Cases started decreasing from the 18th of January gradually to the 24th of January. Although cases were still being seen after the outbreak period, the figures were below the action threshold line Fig. 1.

### Knowledge on malaria

Knowledge on the cause and treatment of malaria was good comparable between both cases and controls. Few respondents from each group (5, 7 % cases and 4, 6 % controls) wrongly stated that either one gets malaria from eating unripe fruits or drinking contaminated water. A few of the participants from each arm wrongly stated the first line treatment for malaria with 10 out of 87 (11, 5 %) cases and 12 out of 87 (13, 8 %) controls stating that chloroquine was still being used in treating malaria. The difference in knowledge of transmission and treatment of malaria between cases and controls was not statistically significant.

Knowledge about malaria was further assessed by asking study participants about symptoms of malaria and methods of preventing and controlling malaria. The most commonly stated symptom by both cases and controls was headache (85, 1 and 92 % respectively). Sleeping under an insecticidal treated net was the most common method of preventing malaria stated by both cases and controls (92 and 98, 8 % respectively.)

### Factors associated with contracting malaria

The study went on to single out risk factors associated with contracting malaria during the outbreak period. Table 2 shows the relationship between studied risk factors and getting infected with malaria. Sleeping under a treated mosquito net and wearing protective clothing when outdoors before dawn or after dusk were found to be a protective factors (OR = 0,272, p value = 0, 0005 and OR = 0,121, p value = 0.00 respectively). Being under the age of 5 years, carrying out activities outdoors and not using repellents increased risk of contracting malaria. These factors were statistically significant.

**Table 2:** Factors associated with contracting malaria in Honde Valley, Mutasa District. 2014

Factor	Cases n (%)	Controls n (%)	OR	95 % CI
Age				
Under 5	9 (10, 3)	1 (1, 1)	9.9	1, 2–80.1
Above 5	78 (89, 7)	86 (98, 9)		
Not using repellents				
Yes	76 (88, 4)	42 (48, 7)	8, 3	3, 8–18, 0
No	10 (11, 6)	45 (51, 7)		
Outdoor activities before dawn and after dusk				
Yes	81 (93, 1)	71 (81, 6)	2, 8	1, 0–7, 6
No	6 (6, 89)	16 (18, 39)		
House sprayed				
Yes	75 (87, 4)	70 (80, 5)	1, 7	0, 8–3, 8
No	11 (12, 6)	17 (19, 5)		
Sleeping under net				
Yes	56 (65, 1)	76 (87, 4)	0, 3	0,1–0,6
No	30 (34, 9)	11 (12, 6)		
Wearing protective when outdoors				
Yes	10 (11, 5)	45 (51, 7)	0, 1	0,1–0,3
No	77 (88, 5)	42 (48, 3)		

Eleven out of eighty-seven cases (16, 2 %) did not receive indoor residual spraying (IRS) compared to 17/87 (19, 5 %) controls. There was however no statistical difference in the risk of contracting malaria between those that had their houses sprayed and those that did not.

### Treatment

All cases that participated in the study were treated according to the national guidelines. On interview, all cases revealed they had a RDT to confirm malaria infection before treatment was given and received Coartemether as treatment. Approximately 50, 6 % accessed treatment from Village Health Workers whilst the remainder were treated at their local clinic. Almost 98 % of cases reported they completed treatment course. Sixty two of the cases (71 %) reported they had received some form of health education together with treatment. The most common subject of health education given was on how to take treatment (70 %) and how to prevent malaria infection (30 %).

Nearly 38 % (33/87) of cases reported having used alternative medicines before seeking healthcare. The most frequently mentioned alternative treatment was ‘holy water’.

## Discussion

Because the area had received optimal malaria control interventions including IRS and LLINs, one naturally expects to realise a significant reduction in cases of malaria. This however is not the case for Honde Valley area.

Contrary to science, a small risk of 1.7 times was noted on those whose houses were sprayed this ‘would be ironic’ finding was not statistically significant. It is however interesting if this result is looked at together with recent bioassay and vector susceptibility study results from Honde Valley by Dr Masendu, a local entomologist (unpublished document) which showed that there was significant vector resistance to the pyrethroids, a chemical used for IRS in Mutasa district. Additionally, a field survey by local entomologists together with the Southern African International Centres of Excellence for Malaria Research (ICEMER) with support from funding partners demonstrated that there has been a change in the species of mosquitoes in the region, with the re- emergence of *Anopheles funestus* a specie known to have an indoor biting and resting characteristic but also be resistant to pyrethroids and carbamates. If the above is to be taken into account, then continuing IRS using chemicals that are currently in use raises the following questions—Are we spraying in vain? Are we throwing scarce resources down the drain? Most importantly are we giving communities especially that of Mutasa District a false sense of protection?

Sleeping under an insecticidal net was found to be a protective factor.

Both local, regional and international papers support this finding [2, 3]. It is interesting to mention that the chemical impregnated in the LLINs distributed in the area under study are pyrethroids- the same chemical group used for IRS. With this in mind one then is keen to know why in this study net use was a significant protective factor from malaria infection when IRS was not a significant protective factor if both used pyrethroids. The only explanation could be that the barrier effect of the net was preventing mosquito bites thus explaining the protective factor of bed nets in this area.

There was a significant eight times risk of getting malaria infection in those that were not using topical mosquito repellents. The community has a low uptake of repellents. Considering that this study showed that almost 86 % of participants survive on a monthly income of less than $100, buying repellents can be considered a luxury. Our study showed that there was a 2, 8 higher risk of getting infected with malaria in individuals who admitted to carrying out activities outdoors before dawn and after dusk. Use of repellents is indicated in areas where outdoor biting is common, rendering best practices like use of LLINs and IRS ineffective. It is well documented that outdoor biting by vectors may increase as a result of either species shifts or behaviour change in response to insecticides use over time [4–7]. Outdoor biting is also common in a sub tropical area where agriculture is an important economic activity. Honde Valley is has a hot and humid climate, with temperatures going as high as 30^°^ C and very high rainfall annually-ideal for vector breeding. There are gravity fed irrigation channels and stretches of wetlands in the area. Covering the 500 square kilometre area, the valley is filled with thick forests and extensive tea, coffee and banana plantations. Commercial farmers employ the local people. Most of the locals also have small ‘plantations’ of their own growing bananas and yams for subsistence and coffee and tea for resale. The people in this area spend most of their time outdoors from dawn to dusk; farming for a living and exposing themselves to the vector. Combining the information and findings from this study, one can deduce that using repellents in Honde Valley will benefit the community.

Use of repellents as a way of preventing infection with malaria has been shown to be effective in a number of studies. Trials carried out in Malaysia and Thailand showed that there was an 83 % reduction [8, 9] in mosquito bites in populations using repellents. A 56 % reduction in transmission rates of *Plasmodium falciparum* was demonstrated in Pakistan amongst households using a soap containing a commonly used chemical in repellents-di-ethyl-N, N-toluamide (DEET) [10]. Cambodia in its battle against malaria 2012 embarked on a 2 year project to test effectiveness of the mass use of topical repellents so their use may be in cooperated into the national strategic anti malaria plan. Results of this experimental study where one arm of the community is LLINs and the other LLINs and a topical repellent have revealed that use of repellents has a significant protective factor in reducing infections. The area where this study is undergoing is characterised by thick forests and farming activities, much like Honde Valley. It is however important to conclude by mentioning that a recent article from a study done in Tanzania shared results that showed that high coverage of repellent use could significantly reduce man-vector contact but incomplete coverage in any particular area will result in a diversion of the vector to the area not covered by repellents [11]. It is thus important that if repellents are to be considered for vector control, maximal coverage should be targeted for.

Currently, although the benefits of repellent use are known, Zimbabwe has no mosquito repellent policy. If a policy of mass use of repellents is adopted will it yield the same results as those in other countries? Will it be a cost effective intervention? Will repellents be acceptable by the community? These and other questions can only be answered though further local studies.

## Conclusions

Evidence from this outbreak investigation has shown that the outbreak in Honde Valley in Mutasa District was a result of possible emergence of resistance to pyrethroids used in IRS in the area, less than optimum use of LLINs and increased outdoor activities by the community before dawn and after dusk with low utilisation of repellents. We thus recommend that the NMCP carry out malaria vector insecticide susceptibility studies in the District and if resistance to pyrethroids is confirmed, the MOHCC should consider changing to a more efficacious insecticide as guided by the WHO document on guiding principles on insecticidal resistance management. Targeted health education messages can be pointed to use of mosquito repellents when the community is outdoors before dawn and after dusk and to use of LLINs consistently.

## Abbreviations

IRS: indoor residual spraying
LLINs: long lasting insecticidal nets
WHO: World Health Organisation
NMCP: National Malaria Control Program
MOHCC: Ministry of Health and Child Care
CFR: case fatality rate
DEET: di-ethyl-N–N-toluamide
