# Erratum to: Factors associated with malaria infection in Honde valley, Mutasa district, Zimbabwe, 2014: a case control study

**DOI:** 10.1186/s13104-017-2745-z

**Published:** 2017-08-24

**Authors:** Norma Mugwagwa, Joseph Mberikunashe, Notion Tafara Gombe, Mufuta Tshimanga, Donewell Bangure, More Mungati

**Affiliations:** 1 Department of Microbiology, Mbarara University of Science and Technology, P.O. Box 1410, Mbarara, Uganda; 2 Epicentre Mbarara Research Centre, P. O Box 1956, Mbarara, Uganda

## Erratum to: BMC Res Notes (2015) 8:829 DOI 10.1186/s13104-015-1831-3

Following publication of the original article [1], one of the authors reported that her name had been captured incorrectly as Noma Mugwagwa, instead of Norma Mugwagwa.

The original article has been corrected.
